# Study on Microstructure Characteristics of Axially Braided Carbon/Carbon Composites Based on SEM and Micro-CT

**DOI:** 10.3390/ma13061414

**Published:** 2020-03-20

**Authors:** Chunguang Wang, Min Tang, Weikai Liu, Tao Zhu

**Affiliations:** 1 College of Electrical and Control Engineering, Shaanxi University of Science & Technology, Xi’an 710021, China; 2 The Fourth Academy of CASC, Xi′an 710025, China; leegoop@126.com (M.T.); wakenliu@sina.com (W.L.); 3 The 41st Institute, The Fourth Academy of CASC, Xi’an 710025, China; zhutao100200@163.com

**Keywords:** Axially braided C/C composites, mesoscopic and micro, three-dimensional and four- directions, crack, porosity

## Abstract

In order to study the microstructure characteristics of an axially braided Carbon/Carbon (C/C) composite, a comprehensive observation and study of the mesoscopic and microstructure characteristics of an axially braided C/C composite is conducted. Scanning electron microscopy and Micro-CT were used to obtain the microstructure characteristics and distribution rules of the axially braided C/C composite material. The physical model of the material and the geometric model of the representative unit were established. At the same time, the characteristics of this kind of material are also obtained. The microstructure characteristics show that the axially braided C/C composite is a polymer with cracks and pores of different sizes, which is a three-dimensional and four-directions carbon fiber braided body as the reinforcing phase and pitch carbon as the reinforcing matrix. The microcosmic data obtained in this chapter is the basis for carrying out material property prediction and qualitative comparison of macro performance.

## 1. Introduction

The Carbon/Carbon (C/C) composite material, namely carbon fiber reinforced carbon matrix composite material, is a structural composite material with a reinforcing property and a matrix phase composed of pure carbon with special properties developed in the late 1950s [1,2]. It has a series of excellent properties such as high specific strength, good thermal stability, abrasion resistance, and anti-ablation, especially its mechanical properties. For the strength of this material does not decrease with the increase of temperature, so it has been widely used in aviation and aerospace 18 [3,4]. Because it is a cross-scale structural material, the micromechanical characteristics of the material are the basis for studying the macroscopic properties of the material. To accurately grasp its mechanical properties, a comprehensive analysis of its cross-scale internal structure is required [5].

For decades, many scholars have used a variety of different testing methods, testing equipment and analysis methods to study the micro-structure of C/C composite materials. Among them, SEM plays an important role in the observation and analysis of C/C composites [6], and can be used to analyze the structure of the fiber monofilaments inside the fiber bundle, the surface state of the C/C composites, and the shape of the fractured section. In addition, the crack growth of C/C composites under load conditions can also be observed in real time with a scanning electron microscope [7]. In addition, the observation accuracy of the high-resolution Micro-CT system has been developed to the micron or even nanometer level, making it widely used in the observation and three-dimensional reconstruction of the internal microstructure of multidirectional braided composite materials to obtain the in-situ microstructure of the material. Therefore, it is expected to analyze the effective macroscopic properties of woven composites based on the microstructure [8,9]. Comprehensive literature analysis found that [10], domestic and foreign countries have made certain achievements in the study of the microstructure characteristics of axially braided C/C composite materials, and have gained more instructive experience, but these studies usually only focus on a certain level. Observation and analysis of local structures such as local microstructures did not completely and systematically analyze the mesoscopic and micro structure characteristics of C/C composites [11,12,13,14,15,16].

The carbon–carbon composites with similar structure mainly include direct braided and puncture C/C composites, wound braided C/C composites, three-dimensional four-way braided C/C composites, multidimensional braided C/C composites, and needled carbon/carbon composites. The main results of the researches on its microscopic properties carried out by scholars are shown in Table 1.

From the above research, it can be found that the microscopic properties of C/C composites have been studied a lot, but the microscopic characteristics of axially braided C/C composites have been seldom studied. In order to study the properties of materials, it is urgent to characterize their microstructure. This paper is an experimental study on axially braided C/C composite materials. In this paper, a comprehensive analysis of the mesoscopic and micro structure characteristics of the axially braided C/C composites is performed. Scanning electron microscopy was used to observe and analyze the surface microstructure of the enhancement phase, matrix phase, and interface of the material, and high-resolution Micro-CT was used to observe and analyze the internal microstructure. In addition, according to the observation results, the scale range of the microstructure characteristics of the unit cell model of the axially braided C/C composite material is obtained. The microscopic data obtained in this paper is the basis for carrying out material property prediction and qualitative comparison of macroscopic properties.

## 2. Materials and Methods

### 2.1. Axially Braided C/C composite Material

**Figure 1:**
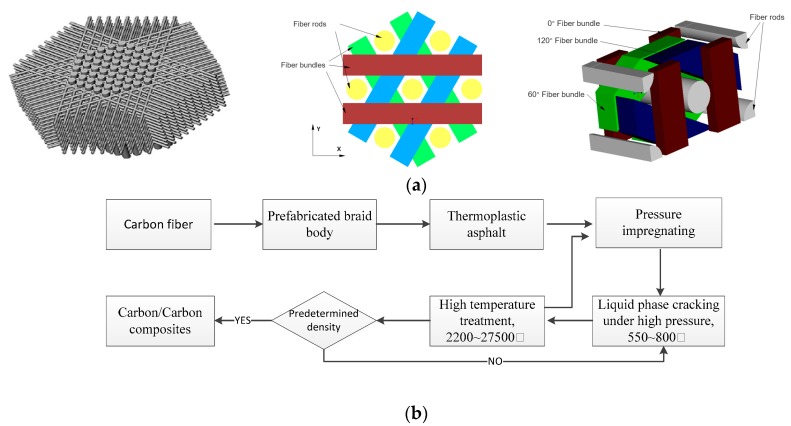
The braided form and manufacturing procedure of axially braided C/C composite material (**a**) the braided form of axially braided C/C composite material.The intersecting directions of the fiber rod and fiber bundle are shown in the figure; (**b**) the manufacturing procedure of axially braided C/C composite material.

The braided structure of axially braided C/C composites is shown in Figure 1a. The material consists of a pultruded rigid carbon fiber rod that forms an axial reinforcement network and is pre-woven from a soft carbon fiber bundle. The fiber rods are arranged in an equilateral triangle in the axial direction, and the fiber bundles are successively increased through the 0°, 60°, and 120° channels of cambium formed by the fiber rods, and so on until the required size of prefabricated braid body is formed. The prefabricated braid body were prepared by asphalt impregnation, carbonization, densification, and high temperature treatment. Its manufacturing procedure is shown in Figure 1b. The minimum element of this pre-texture is symmetric along the axis and its braiding thickness accumulates along the axis, so it is called axially braided C/C composite material.

### 2.2. Sample Preparation and Instrument

#### 2.2.1. The Experimental Materials

**Figure 2:**
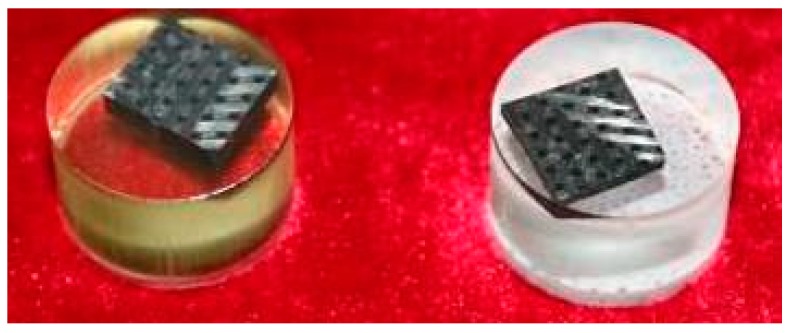
Test samples after curing.

In this paper, the axially braided C/C composite material produced by China Academy of Aerospace Power Technology (Xi’an, China) was taken as the object of study, and its microstructure was observed. A total of 100 samples were observed to determine the common characteristics of the material, so as to give a physical model. Test samples after curing is shown in Figure 2.

#### 2.2.2. Low Damage Sample Preparation Method

The axially braided C/C composite material is a kind of brittle material. Before observing its microscopic characteristics, it should be noted that the material should be sampled with low damage. The most common method is to cut, impregnate and polish the C/C material samples, and make them into observable experimental samples. Resin impregnation and fluorescent staining were used to improve the authenticity of observation of microstructure characteristics.

#### 2.2.3. Experimental Equipment and Operative Conditions

In this paper, high resolution SEM equipment and Micro-CT system are used for microstructure observation experiments.

The model of the SEM equipment is SIGMA 300. Its manufacturer is Carl Zeiss of Germany (Oberkochen, Germany). Main parameters of SEM equipment selected in this paper: sample size diameter—60 mm, height—45 mm, maximum magnification rate—150,000×, and maximum resolution—5 nm.

The model of the Micro-CT system is nanovoxel-5000. Its manufacturer is San Ying Instruments Co., LTD of China (Tianjin, China). The main parameters of the Micro-CT system selected in this paper are: radial field diameter ≥36 mm, image reconstruction pixel—4.5 μm, and the highest reconstruction resolution up to 2.3 μm.

The axially braided C/C composites used in the experiment are the axially braided C/C composites described in Section 2.2.1. The axially braided C/C composite material was processed into 1 × 1 × 0.2 cm^3^ thin sections, and the surface was polished and polished. With alcohol as solvent, ultrasonic cleaning equipment is used to clean the surface dirt and powder formed during processing. The ambient temperature of the test is 20 °C. In order to ensure the accuracy of the measurement results, it is carried out on a dust-free test platform.

### 2.3. Dimensions and Representative Units of Axially Braided C/C Composites

**Figure 3:**
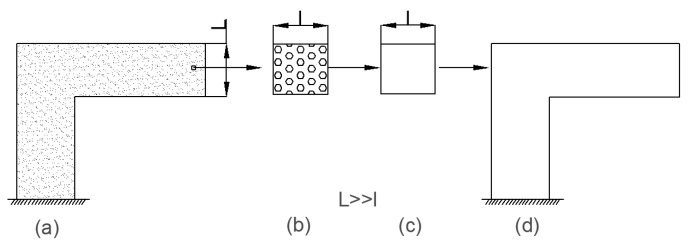
Composite structure, representative unit and equivalent structure (The diagram illustrates the relationship between the three). (**a**) Composite structure; (**b**) The local element; (**c**) Representative volume element; (**d**) The equivalent structure after homogenization.

**Figure 4:**
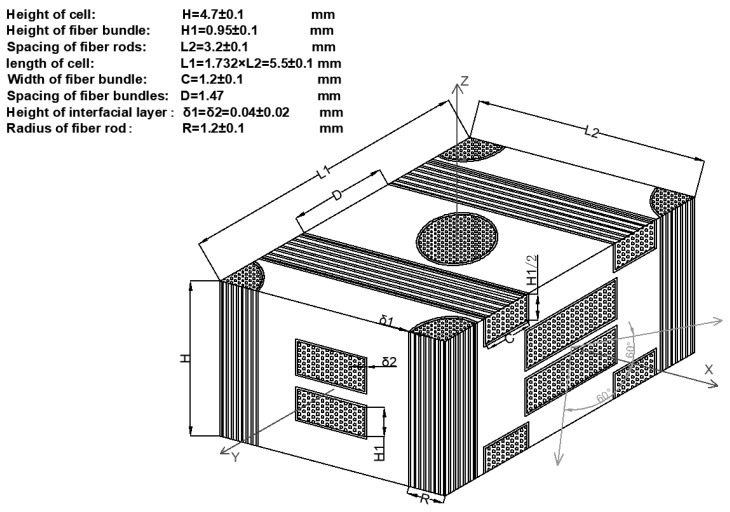
Representative volume element (RVE) unit of axially braided C/C composite (This is data obtained from the observation of 100 samples.)

In order to study the performance of the axially braided C/C composite material, the concept of representative volume element (RVE) is first introduced. The concept is shown in Figure 3. For general composite materials, the scale is in the order of micrometers, and the structure scale is in the order of centimeters to meters, which represents the unit is in the order of millimeters. RVE scale and inclusion scale satisfy L >> l >> A. A represents the size of inclusions (fibers). For axially braided C/C composites, the microscale is in the micrometer to millimeter range (μm ~ mm), which mainly involves pores, cracks, fiber monofilaments, interfaces, and matrix in fiber rods (bundles). Its mesoscopic is in the millimeter to centimeter range (mm ~ cm), mainly including fiber bundles, fiber rods, matrix and interfaces between fiber rods (bundles). The RVE unit of the axially braided C/C composite is a rectangular parallelepiped structure composed of fiber rods, fiber bundles, matrix and interfaces, as shown in Figure 4. It is a repetitive structure composed of numerous representative units.

## 3. Results

The microstructure characteristics of axially braided C/C composites mainly include the geometry of the component phases, the morphology of the component phases, and the dimensions and distribution of the internal fibers, pores, and cracks. These microstructure features can be divided into apparent microstructure features and internal microstructure features.

### 3.1. Surface Structure Characteristics of Axially Braided C/C Composites

**Figure 5:**
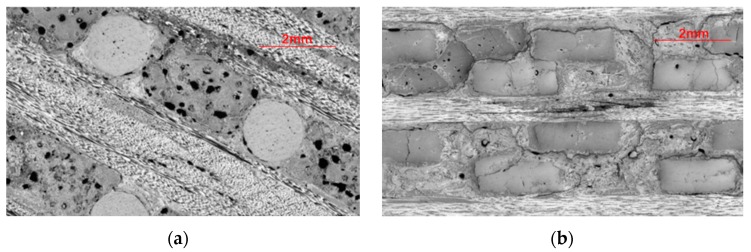

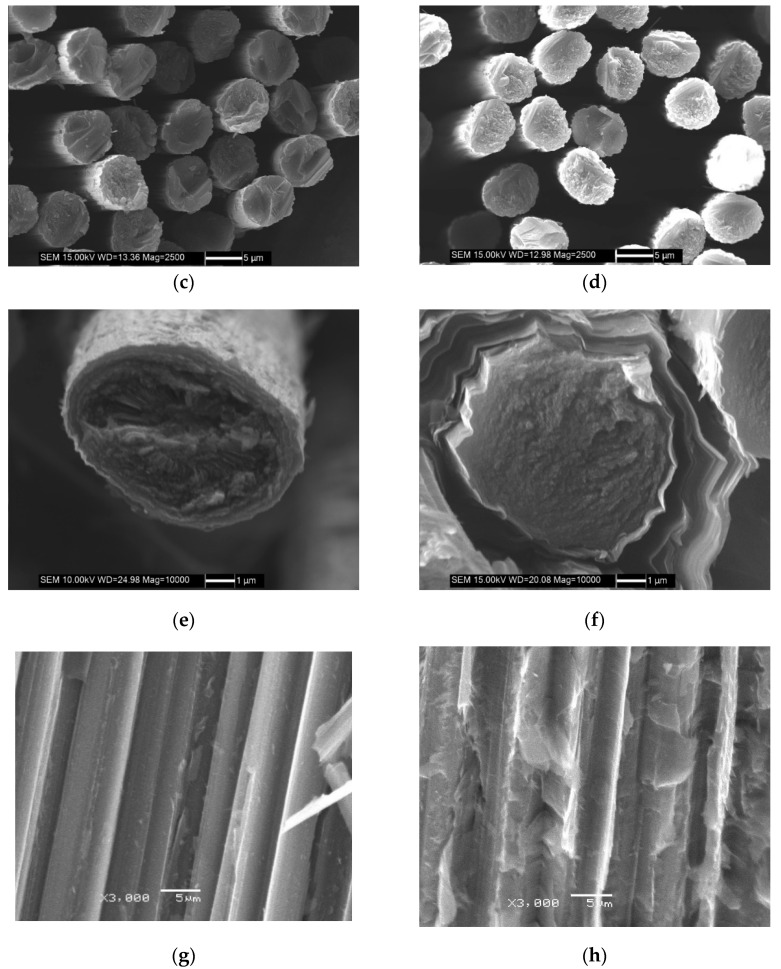
Surface microstructure of the reinforcing phase of axially braided C/C composites. (**a**) Axial micrograph of axially braided C/C composites by SEM; (**b**) Radial micrograph of axially braided C/C composites by SEM; (**c**) Micrograph of the fiber rod end surface by SEM; (**d**) Micrograph of the fiber bundle end surface by SEM; (**e**) Fiber morphology in the reinforcement phase by SEM; (**f**) Interface morphology between fibers and matrix by SEM; (**g**) Cross-section morphology of fiber rod by SEM; and (**h**) Cross-section morphology of fiber bundle by SEM.

Figure 5a shows the axial morphology of the axially braided C/C composite material. The axial fiber rods (hereinafter referred to as fiber rods) are arranged in a regular triangle The radial fiber bundles (hereinafter referred to as fiber bundles) pass through the channels between the fiber rods. The gap between the fiber rod and the fiber bundle is filled with pitch charcoal. The gap between the fiber rod and the matrix charcoal is obvious, indicating that the interface between the matrix charcoal and the fiber rod is weak. There are large holes and defects in the matrix charcoal, and the holes are mainly spherical and ellipsoidal. There are microcracks in the fiber bundle. There is a small gap at the interface between the fiber bundle and the matrix. Compared with fiber rods, the interface hole between the fiber bundle and the matrix has fewer holes.

Figure 5b shows the radial micro-morphology of the axially braided C/C composite material. It can be seen that the fiber bundles are arranged regularly and there are cracks inside. The fiber bundle and the fiber bundle are filled with pitch charcoal, and there is a partial gap between the fiber bundle and the matrix, indicating that the fiber bundle and the matrix are weakly bonded at the interface. The matrix charcoal is full of holes and contains cracks.

The braided structure parameters obtained by means of microscopic observation are shown in Table 2.

#### 3.1.1. Surface Microstructure Characteristics of the Reinforcing Phase

Fiber rods and fiber bundles are unidirectional reinforced composite materials with fiber filaments as the reinforcing phase and asphalt charcoal as the matrix phase. The difference between the two is that the fiber rods need to be stretched into a rod shape with a certain diameter after prepreg. The fiber bundle is a combination of fiber filaments, which directly pass through the space channel formed by the fiber rod, and forms a rectangular-shaped reinforcing unit in the composite process.

Figure 5c,d are partial SEM images of the end faces of the fiber rods and fiber bundles. It can be seen from the figure that the fiber distribution positions are random, and the fiber filaments in the fiber bundles are looser than the fiber rods. Under further magnification, it can be more clearly seen that the cross section of the fiber filaments is mainly circular and oval, and the ovality of some materials is large, and the fibers have radial stripes from the core outward (Figure 5e). The outer layer of the fiber is covered with a layer of matrix charcoal, which is a carbon structure that is easy to graphitize. From the interface morphology of the fibers, it can be seen that the matrix connection phase between the fibers is lamellar and oriented along the fiber axis (Figure 5f).

Figure 5g,h are cross-sectional SEM morphologies of fiber rods and fiber bundles. Under the same sample preparation process, there is less matrix inside the fiber rod, and the fibers and fibers are not completely filled with asphalt charcoal and have more pores. The fibers and fibers inside the fiber bundle are basically filled with asphalt charcoal, and the bond between the fiber and the matrix is tighter.

Therefore, the interfacial shear strength between the carbon fiber monofilament and the matrix in the fiber bundle is large. This is because the fiber rod is pultruded from the resin- impregnated fiber bundles, and the asphalt is not easy to infiltrate when the asphalt is impregnated, which results in less asphalt between the fibers and the tight bonding.

**Figure 6:**
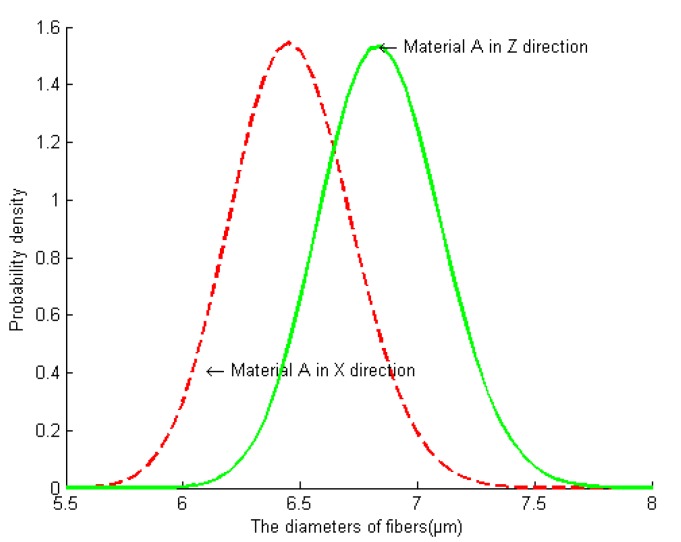
Fiber diameter distribution of axially braided C/C composites in different directions (more than 1000 fibers).

Using Matlab software to program the processing of Figure 5c,d, the fiber grayscale graphs in the figure can be converted into binary graphs to calculate the in-situ size of the fiber filaments. By performing image processing on a certain number of fibers (more than 1000 fibers), the average area and distribution of carbon fibers can be obtained. The carbon fiber diameter distribution can be obtained by making the carbon fiber cross section equivalent to a circle. Figure 6 shows the fiber diameter distribution in different directions of the material. The research shows that the carbon fiber diameter obeys the log–normal distribution. The mean diameter of axial (fiber rod) fiber is 6.46 μm and the variance is 0.04. The mean value of radial (fiber bundle) is 6.84 μm and the variance is 0.038.

Using the same calculation method as the calculation of the area of fiber filaments, processing the fiber rod and fiber bundle images in the material can obtain the true area of the entire fiber rod (fiber bundle). According to the number of fibers contained in the fiber rod (fiber bundle), calculate the total area of all fibers, and divide it by the total area of the fiber rod (fiber bundle) to obtain the volume content of the fiber in the reinforcing phase. Table 3 shows the fiber volume content in fiber rods and fiber bundles. Obviously, the fiber volume content in fiber rods is larger than that in fiber bundles.

Due to the small pores inside the fiber rod, the image method was used to study the pore distribution inside the fiber rod. After testing and analyzing 1000 fiber rods, the porosity distribution in the fiber rods is between 0.043–0.0569, and the shape of the pores is most common in the shape of a triangle. The irregular holes in the fiber rod with any direction are equivalent to a spherical shape. Based on the calculation of 1000 pores, the diameter of the pores in the fiber rod obeys the normal distribution. The average pore diameter is 1.96 μm and the variance is 0.04.

#### 3.1.2. Surface Microstructure Characteristics of the Matrix Phase

**Figure 7:**
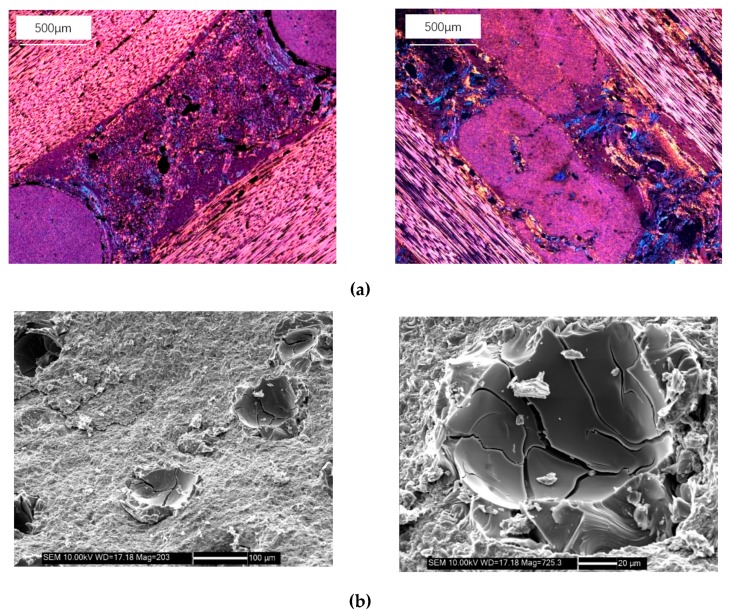
Surface microstructure characteristics of the matrix phase of axially braided C/C composite. **(a)** Micrograph of the carbon matrix phase by SEM and **(b)** morphology of carbon matrix pores by SEM.

Figure 7a shows the micro-morphology of the axial and radial matrix of the axially braided C/C composites. The matrix is filled with pores and cracks, and the pore size distribution is extremely uneven. Further SEM image shows the main morphology of the pores (Figure 7b), which is more common in ellipsoids, with a large number of cracks at the bottom of the pores, reflecting the brittleness of the matrix.

**Figure 8:**
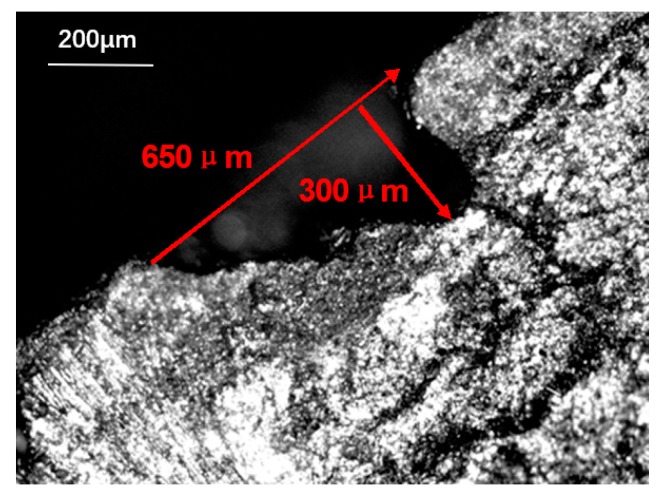
Equivalent test method for pores.

**Figure 9:**
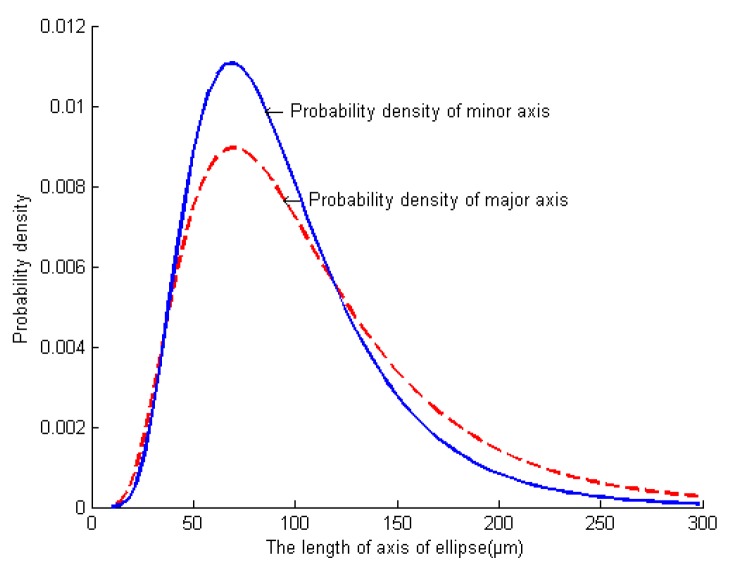
Probability density distribution of matrix pore size.

Porosity and short cracks were taken as the main microstructure characteristics for statistics. They were respectively treated as ellipsoids with different long and short axes. Figure 8 shows the equivalent test method of pores. By measuring the long and short axes of 1000 pores and short cracks on the matrix of different positions on the axially braided C/C composite, it was obtained that both the long and short axes satisfied the log–normal distribution.

(1) $$N(R^{\prime })=\frac{1}{R^{\prime }\sqrt{2\pi \sigma }}\exp\left\{-{\left[\frac{\ln\left(R^{\prime }/R\right)}{\sqrt{2}\sigma }\right]}^{2}\right\}$$

where $R^{\prime }$ is the short axis (long axis) length of the pores. R is the average length of the short axis (long axis). σ is the standard deviation of the logarithm of the length variable. Short semi-axis average length $R_{s}=85.2\mu m$, long semi-axis average length $R_{l}=94.2\mu m$. The short axis standard deviation $\sigma_{s}=0.47$, the long axis standard deviation $\sigma_{l}=0.55$. The probability density curve of the matrix pore size is shown in Figure 9.

#### 3.1.3. Surface Microstructure Characteristics of the Interface

**Figure 10:**
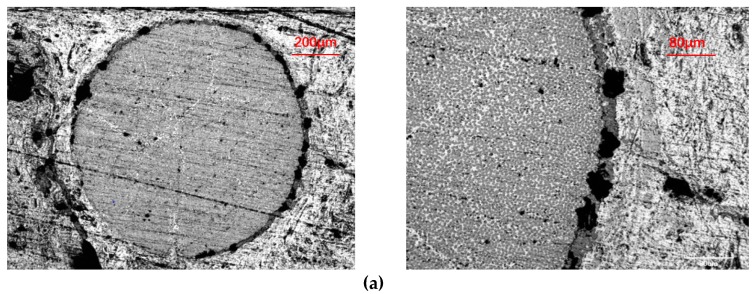

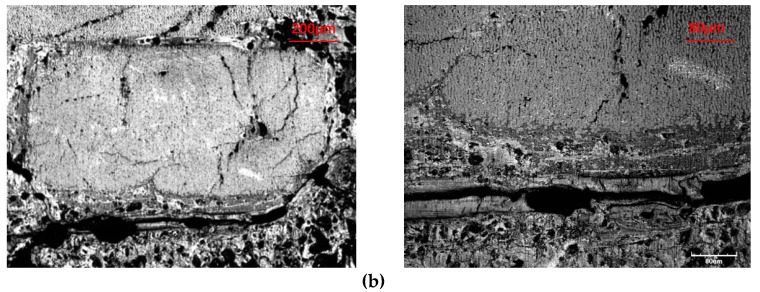
Surface microstructure characteristics of the interface of axially braided C/C composites. (**a**) Microstructure of interface layer of fiber rod by SEM; (**b**) Microstructure of interface layer of fiber bundle by SEM.

It can be seen from Figure 10a,b that between the fiber rod, the fiber bundle and the charcoal matrix, there is a clear interface layer, and there are a large number of holes and delaminating in the interface layer. At the interface of two different fiber bundles, the fiber bundles are not in direct contact, and there is a double-layer interface, that is, an interface layer exists in each fiber bundle. The interface can be regarded as an annular transition region with a certain thickness between the fiber bundle and the matrix charcoal, with a thickness of about 20 μm.

The same treatment method as the matrix phase was used to calculate the size of the 1000 pores and short cracks in the interface phase. The scale distribution was also in accordance with the lognormal distribution of formula (1). Short semi-axis average length $R_{s}=50.6\;\mu m$, long semi-axis average length $R_{s}=50.6\;\mu m$. The short semi-axis standard deviation is $\sigma_{s}=0.42$, long semi-axis standard deviation is $\sigma_{l}=0.51$.

### 3.2. Internal Mesoscopic/Microstructure Characteristics

**Figure 11:**
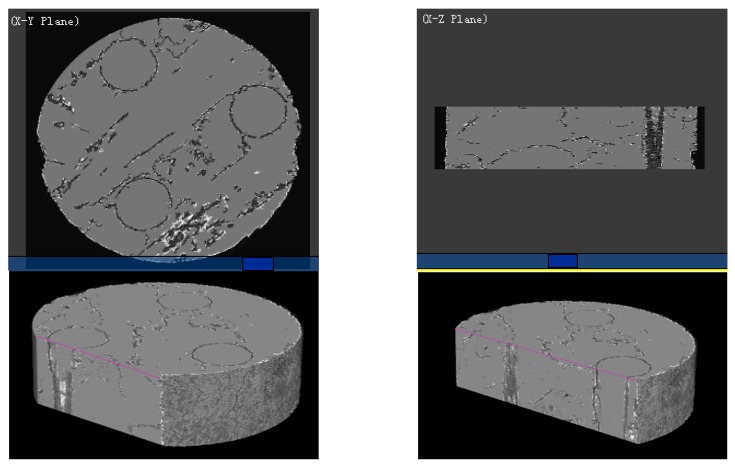
Sectional view of the physical model of the axially braided C/C composites microstructure feature by Micro-CT.

Because the microscope and scanning electron microscope can only observe a certain surface of the material, and the sample preparation method may damage the microstructure of the material, thereby affecting the in-situ nature of the observed area. Compared with the traditional method, the micro-CT system can observe the microstructure information at any level inside the material, and obtain a non-destructive, complete and in-situ internal microstructure. In this paper, the micro-CT system was used to scan the axially braided C/C composites and perform 3D reconstruction. Reconstructed the pre-processed 1000 images, and a physical model of the internal microstructure of the axially braided C/C composite material was obtained (Figure 11). The physical model can be cut at any position to obtain the microstructure characteristics of any part inside the composite material. It can be seen that the pore distribution of axially braided C/C composites has random and non-uniform characteristics. The interface between the fiber rod/matrix and fiber bundle/matrix is full of pores, showing a weakly bonded morphology. The conclusions reached are the same.

**Figure 12:**
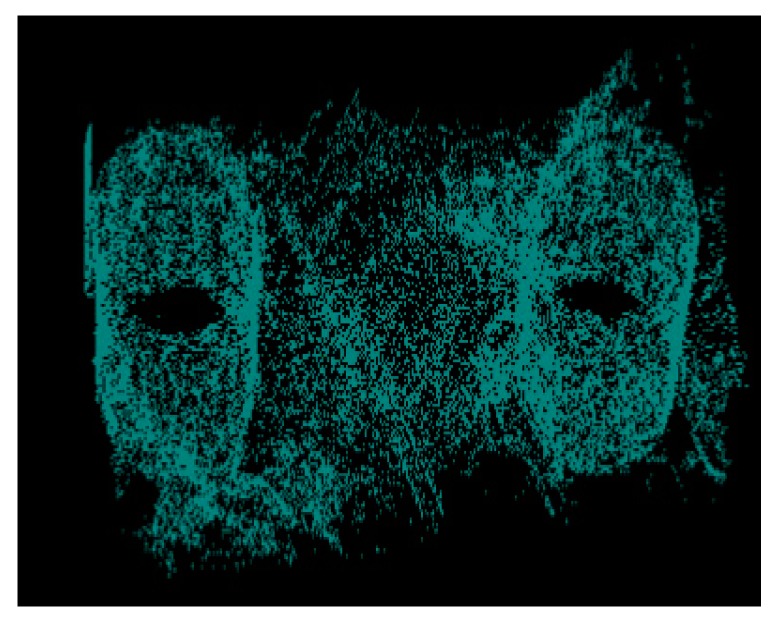
Three-dimensional spatial structure of the axially braided C/C composites interface by Micro-CT.

**Figure 13:**
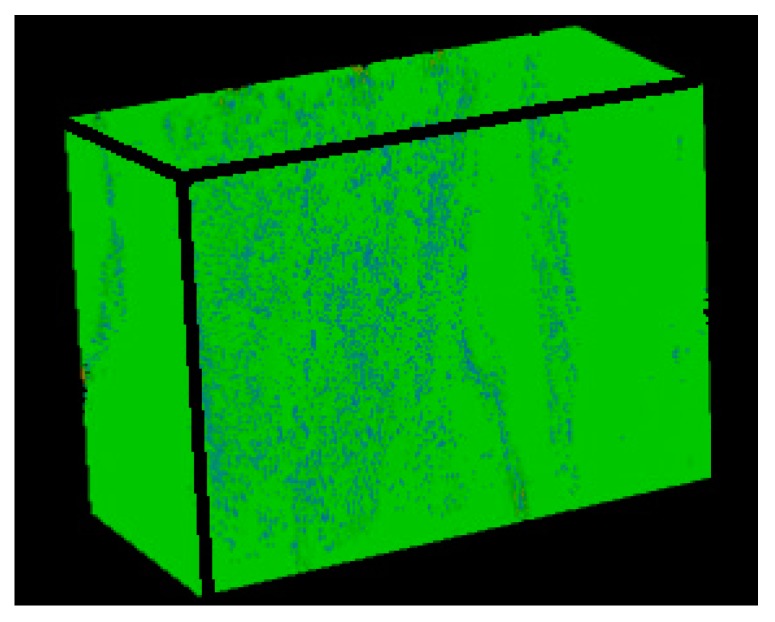
Three-dimensional structure of axially braided C/C composite matrix charcoal by Micro-CT.

By extracting the microstructure characteristics of the interface, the three-dimensional spatial structure is shown in Figure 12, and the analysis shows that the interface has a porosity between 0.254 and 0.3, with a normal average porosity of 0.27 and a variance of 0.017. The three-dimensional spatial structure of the matrix obtained by the same image processing method is shown in Figure 13. The matrix has fewer pores than the interface. The porosity distribution of the matrix at different positions is between 0.0327 and 0.04. Its normal mean is 0.036 and the variance is 0.015.

## 4. Discussion

Based on the above research and analysis, it can be found that the axially braided C/C composite is a polymer with cracks and pores of different scales, with three-dimensional four-direction carbon fiber braiding as the reinforcing phase and asphalt carbon as the reinforcing matrix. It is both a material and a multi-level structure, which can be divided into three levels for research: macro, meso, and micro levels. The macro level corresponds to the repeatable structure of the axially braided C/C composite material. The mesoscopic level corresponds to the internal component materials (fiber bundles, fiber rod, and matrix) and the interface layer of the axially braided C/C composite material. The micro level corresponds to the component materials and the internal microstructure of the interface layer. The physical model of the axially braided C/C composite microstructure is shown in Figure 4, and its characteristics are described as follows:

1. The fiber rods in the Z-direction of axially braided C/C composites are Φ1.2 mm. The rods are arranged in a regular triangle. The center-to-center distance between the rods is about 3.2 mm. The fiber bundles are tiled in clearance of fiber rod, and the fiber bundles form 60° with each other, and a period of three layers of fibers with a distance of about 2.7 mm. The other parts of the composite material are filled with matrix charcoal to ensure the overall stability of the composite material. The carbon fiber filaments diameter obeys the log-normal distribution. The average axial fiber filaments diameter is 6.46 μm, and the variance is 0.04. The radial average is 6.84 μm, and the variance is 0.038.
2. The interface between fiber rods (fiber bundles) and matrix charcoal in C/C composites is a weak bond, there are obvious pores between fiber rods (bundles) and matrix charcoal, and they are basically penetrating. The matrix charcoal Existing between carbon fiber rods, between layers of fibers, and in XY-direction fiber bundles. The pores and cracks are randomly distributed in the matrix charcoal.
3. The axially braided C/C composite material is a porous material, and the pores are mainly distributed in the matrix charcoal, the interface, and the fiber rod (fiber bundles). The overall porosity of the material is between 0.045 and 0.055. The pore shape of the fiber rod is nearly triangular, and the porosity is about 0.043–0.0569. The interface porosity is between 0.254 and 0.3, the normal mean is 0.27, and the variance is 0.017. The porosity in the matrix is between 0.0327 and 0.04, which is normal mean of 0.036 and a variance of 0.015.
4. The pore size distribution of axially braided C/C composites is concentrated in three areas: macro pore areas larger than 10μm, mesoscopic pore areas 0.1–10 μm, and micro pore areas smaller than 0.1 μm. The largest proportion of macro pores greater than 90 μm is the largest, above 0.7. The mesoscopic pores between 10 and 90 μm account for approximately 0.2–0.25, small pores between 0.1 and 10μm account for 0.03–0.05, and the micro pores little than 0.1 μm account for the smallest proportion, under 0.01. The macro pores and mesoscopic pores mainly exist in the matrix charcoal phase and interface phase of the material, while the small and micro pores mainly exist in the fiber rods and fiber bundles of the material. The pores in the fiber rod can be equivalent to a spherical shape, and the pore diameter obeys a normal distribution. The average value is 1.96 μm, and the variance is 0.04. The pores in the matrix and the interface can be regarded as ellipsoids with different long and short axes, and their sizes obey the log-normal distribution. The average sizes of the long semi axes corresponding to the pores in the matrix and interface are 94.2 μm and 138.8 μm. The variances are 0.55 and 0.51. The average sizes of the short semi axes corresponding to the pores in the matrix and interface are 85.2 μm and 50.6 μm. The variances are 0.47 and 0.42.

Literature [16,17,18,19,20,21,22] has made some achievements in the observation of microstructure characteristics and the test of properties of C/C composites, from which more instructive experience has been gained. However, in terms of the experimental study of microstructure characteristics, although some observation work has been carried out on the microstructure characteristics, these studies usually only analyze the local structure at a certain level, and do not completely and systematically analyze the microstructure characteristics of axially braided C/C composites. In particular, the influence of microstructure characteristics on macroscopic structure is very important. For example, braided spacing, fiber rod and fiber bundle size, fiber volume fraction, matrix pore content, interface performance, etc., have important effects on the macroscopic properties of materials. However, due to the variety and complexity of microstructure characteristics of materials, especially the wide distribution of characteristic parameters such as porosity, crack, pore mean, and pore shape, it is difficult to establish a comprehensive relationship between microstructure characteristics and macroscopic performance with limited experimental data. In this paper, the microstructure characteristics of axially braided C/C composites were observed in detail, and the microstructure characteristics of the materials were obtained systematically. The research results are helpful for the performance analysis and evaluation of axially braided C/C composites, and have important practical value.

## 5. Conclusions

In this paper, through quantitative observation of microstructure characteristics, quantitative characterization data of micromorphology, microstructure and internal microstructure of axially braided C/C composites were obtained. The research shows that the axially braided C/C composite is a polymer with cracks and pores of different sizes, with three-dimensional four-direction carbon fiber braid as the reinforcing phase, and asphalt charcoal as the reinforcing matrix. Axially braided C/C composite can be viewed macroscopically as a periodic arrangement of cuboids with a certain size. Its microstructure features have a high degree of non-uniformity, and the proportion of pores and cracks in different regions and the scale are different. The shape of pores is mainly ellipsoid, and the crack can also be regarded as an ellipsoid with a large ratio of long and short axes, and its scale probability density functions are all log-normally distributed.

Based on the results of the above research, a physical model of the axially braided C/C composite was established, and the standard microstructure characteristics of the axially braided C/C composites were established. Model data and input parameters were provided for predicting the macro mechanical properties of the material. A fulcrum of correlation between microstructure and macro mechanical properties has been established.

## Figures and Tables

**Table 1:** Research progress on microstructure characteristics of C/C composites.

Name of Material	Dimensions	Forms	Research Carried Out
Direct braided and puncture C/C composites	4D	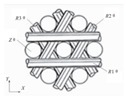	The microstructure was analyzed by SEM, and the parameters of fiber rod diameter and interface defects were studied [17].
Wound braided C/C composites	2D/3D	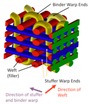	A study on the correlation between weaving technology and performance was conducted by Micro-CT [18].
Three-dimensional four-way braided C/C composites	4D	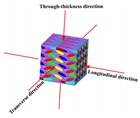	Progressive damage analysis under bending load was carried out and crack propagation was observed by SEM [19].
Multidimensional braided C/C composites	nD	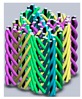	A microscopic model was established to analyze the elastic properties and damage properties of the material [20].
Needled carbon/carbon composites	2.5D	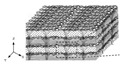	The effects of acupuncture techniques and inclusions on mechanical properties were studied. Numerical prediction of performance based on SEM [21,22,23]

**Table 2:** Axially braided C/C composite material braided structure parameters (The data obtained from the observation of 100 samples).

Fiber Rod Diameter	Fiber Bundle Section Size	Fiber Rod Braid Spacing	Fiber Bundle Monolayer Height	Interfacial Height
1.2 ± 0.1 mm	1.19 × 0.95 mm	3.2 ± 0.1 mm	0.95 ± 0.05 mm	0.02 ± 0.01 mm

**Table 3:** Volume content of fiber reinforced phase in axially braided C/C composites (The data obtained from the observation of 100 samples).

Item	Area (mm^2^)	Total Fiber area (mm^2^)	Volume Content (%)
Fiber rod	1.155	0.7884	68.26
Fiber bundle	1.060	0.664	62.64

## References

[1] Zhang C., Xu X. (2013). Finite Element Analysis of 3D Braided Composites Based on Three Unit-cells Models. Compos. Struct..

[2] Gideon R.K., Sun B., Gu B. (2016). Mechanical behaviors of four-step 1 × 1 braided carbon/epoxy three-dimensional composite tubes under axial compression loading. Polym. Compos..

[3] Gong L.-D., Shen X.-L. (2015). Mesoscopic modeling of three-dimensional four-directional braided composites using energy method. J. Aerosp. Power.

[4] Xu K., Qian X. (2015). Microstructure Analysis and Multi-Unit Cell Model of Three Dimensionally Four-Directional Braided Composites. Appl. Compos. Mater..

[5] Gou J.-J., Fang W.-Z., Dai Y.-J., Li S., Tao W.-Q. (2017). Multi-size Unit Cells to Predict Effective Thermal Conductivities of 3D Four-directional Braided Composites. Compos. Struct..

[6] Shokrieh M.M., Mazloomi M.S. (2012). A new analytical model for calculation of stiffness of three-dimensional four-directional braided composites. Compos. Struct..

[7] Zhang F., Wan Y.M., Gu B.H., Sun B. (2015). Impact Compressive Behavior and Failure Modes of Four-step Three-dimensional Braided Composites-based Meso-structure Model. Int. J. Damage Mech..

[8] Lang F., Zhu J., Li Y., Pan J., Jiangn A., Yang S., Xing Y. (2019). Characterization of the interfacial meso-mechanical properties of composites using the fiber push-out under SEM combing with electron beam moiré method. Acta Mater. Compos. Sin..

[9] Gao B., Tang M., Yang Y., Shi H. (2011). Mechanical experiment of 4D in-plain C/C composites. Acta Mater. Compos. Sin..

[10] Zhai J. (2018). Investigation of Mechanical Properties of 3D Braided Composites Based on Multi-scale Theory. Ph.D. Thesis.

[11] Wang C., Xu G. (2019). The analytical method to compute the strain on the soft PSD in SRM. Int. J. Aerosp. Eng..

[12] Wu S., Liu Y., Ge Y. (2016). Surface structures of PAN-based carbon fibers and their influences on the interface formation and mechanical properties of carbon-carbon composites. Compos. Part A.

[13] Ya J., Liu Z., Wang Y. (2017). Micro-CT Characterization on the Meso-Structure of Three-Dimensional Full Five-Directional Braided Composite. Appl. Compos. Mater..

[14] Shi Y., Lu Y., Ni Z., Zhao L., Li Z., Xiong D.-B., Zou J., Guo Q. (2020). Correlation Between Microstructural Architecture and Mechanical Behavior of Single-Walled Carbon Nanotube-Aluminum Composites. Metall. Mater. Trans..

[15] Turner P., Liu T., Zeng X. (2016). Collapse of 3D orthogonal woven carbon fibre composites under in-plane tension/compression and out-of-plane bending. Compos. Struct..

[16] Yuan J.-Y. (2019). Research on Shear Properties of 3D Carbon-Epoxy Woven Composites. Master’s Thesis.

[17] Xu C.H., Xu D.-S., Song L.-Y., Xu K. (2013). Microstructure characterization from X-ray micro-tomography and tensile failure mechanism of 4D in-plane carbon/carbon composites. J. Solid Rocket Technol..

[18] Goud V., Singh D., Ramasamya A., Das A., Kalyanasundaram D. (2020). Investigation of the mechanical performance of carbon/polypropylene 2D and 3D woven composites manufactured through multi-step impregnation processes. Compos. Part A.

[19] Zhang P.-F., Zhou W., Yin H.-F., Shang Y.J. (2019). Progressive damage analysis of three-dimensional braided composites under flexural load by micro-CT and acoustic emission. Compos. Struct..

[20] Li L., Aliabadi M.H. (2019). Elastic property prediction and damage mechanics analysis of 3D braided composite. Theoret. Appl. Fract. Mech..

[21] Zou J.-J. (2019). Study on mechanical properties of carbon/carbon composite structure based on needled technology. Master’s Thesis.

[22] Zhang P. (2016). Microstructure Modeling and Prediction of Effective Properties of 3D Needled C/C Composites. Master’s Thesis.

[23] Liu W.-K., He G.-Q. (2014). Storage life of silicone rubber sealing ring used in solid rocket motor. Chin. J. Aeronaut..

